# Caring for refugees and asylum seekers in Canada: Early experiences and comprehensive global health training for medical students

**DOI:** 10.36834/cmej.69677

**Published:** 2020-12-07

**Authors:** Kate Merritt, Kevin Pottie

**Affiliations:** 1 Faculty of Medicine, University of Ottawa, Ontario, Canada; 2 Bruyère Research Institute, Ontario, Canada; 3 Departments of Family Medicine & School of Epidemiology and Public Health, University of Ottawa, Ontario, Canada

The Lancet Commission on Migration and Health provides a reminder that racism remains prevalent and that discrimination is a key barrier for refugees and asylum seekers to obtain adequate healthcare.^1^ Inequitable access to care highlights an important role for socially accountable^2^ physicians, specifically in refugee and asylum seeker healthcare. Pragmatic and sustainable change in care provision may come from medical students being able to engage in meaningful experiences caring for refugee populations. Medical schools have an opportunity and an obligation to support experiential learning opportunities for students early in their careers, in order to help prepare them to care for marginalized populations, such as refugees and asylum seekers. ^3^

Practicing physicians are often reluctant to see refugee patients.^4,5^ Commonly cited reasons for this include a physician’s lack of knowledge with refugee health, as well as anticipated communication challenges. Indeed, medical students receive sparse training in cultural competency^6^ and often lack the capacity to work effectively with interpreters. Similarly, physicians may be concerned about the medical legal risks of serving patients with potentially complex health needs, and may be apprehensive about possibly falling behind in clinic and losing income. We do not deny the existence of these and other challenges, but we propose that through meaningful experiences, students can acquire the knowledge and skills to contribute to global healthcare and appreciate the unique lived experiences of their refugee patients. Improving knowledge and comfort working with refugees, allows students to more easily recognize the benefits associated with treating this particular population; i.e., sense of usefulness or involvement in interesting medical cases.^4^ To help address this educational gap in social accountability, the Canadian Collaboration for Immigrant and Refugee Health (CCIRH; www.ccirhken.ca) has developed a global health curriculum for over 2000 medical students. This online curriculum helps medical students learn evidence-based refugee health guidelines and establishes opportunities for students to work with refugees using a community service learning (CSL) approach.^7^

Launched in 2013, the CCIRH established innovative, online Refugee Health Training Modules (termed ‘E-Learning’)^8^ framed around the seven core CanMed competencies. These modules were developed using evidence-based guidelines^9^ and demonstrate how each of the CanMed roles (Communicator, Medical Expert, Scholar, Advocate, Professional, Collaborator and Leader) can be applied to refugee patient care. For example, throughout the online Communicator module, students learn the guiding principles for effectively working with medical interpreters. Similarly, as a Medical Expert, students learn about cultural competency, working in resource-limited settings and principles of prevention in a global health context. Case studies were also designed to challenge students to develop an approach to applying global health competencies in real-life scenarios. Following completion of the modules, students were assessed on each global health competency with an online formative quiz.

The E-Learning modules were built on the premise that the more experienced and educated medical students are in culturally sensitive care, the more comfortable they will be in treating culturally diverse populations as the students transition into practice. With the notion that familiarity breeds comfort and curiosity, we subsequently introduced a CSL model at the University of Ottawa. After completing the online E-Learning modules, students integrated their knowledge by working directly with a newly arriving refugee family in their community. Primary-care physicians with expertise in global health acted as supervisors for the CSL program and were accessible to both students and refugee families if additional support was required. Furthermore, students participated in the CSL program for a minimum of 30 hours throughout the academic year. During this time, students provided mentorship based on the personalized needs of the particular family that they were paired with. For example, this could include providing a range of services such as academic support (i.e., assisting with school enrollment or homework) or practical guidance (i.e., shopping for groceries or applying for a job). Although the student-refugee pairing was not focused on medical or physical health, the interpersonal relationships proved to be mutually valuable; for example, newly arriving refugees received support and mentorship, while students practiced cross cultural communication. This novel training program won the University of Ottawa’s Alex Trebek Innovation Award in 2015 and received significant acclamation from both students and refugee families. Student reflections emphasized that the program not only helped to teach them about the barriers refugees face during the resettlement period, but also highlighted the many similarities and interests shared between the students and refugees. For instance, students bonded with one family by helping set up their Netflix connection, while another student taught a family how to make hamburger, and another student used social media to connect with refugee youth.

Older refugees and asylum seekers often face increased vulnerability from a natural decline in their physical and cognitive health, while also being challenged with cultural and language barriers.^10^ Given the distinct needs of elderly refugees, we suggest the importance of global health education being comprehensive and inclusive of refugees of *all* ages. Consequently, since the initial development of our online E-Learning modules, we have added specific objectives for elderly refugee health, including: culturally sensitive palliative care, trauma-informed care of the elderly and polypharmacy in comorbid older refugees.^10,11^ Creating distinct learning objectives for elder global health will help ensure medical students are being trained on cultural *and* age appropriate care. Similar to our original CSL program, we intend to introduce students to older refugees through a community program. Future initiatives will also focus on developing E-learning modules that include other marginalized refugee populations that students may encounter, including LGBTQ+ and those experiencing homelessness. It is our intent that this training will help improve comfort levels of medical students and allow them to grow an appreciation for the rewards and challenges of working with refugees. In turn, we believe that this approach will help foster the development of socially accountable physicians.

The success of the CSL program has spread to 18 universities in Canada, the American University of Beirut in Lebanon and Limerick University in Ireland. Working with refugees in the community has been transformative for medical students and supports students in being better prepared to meet the social and health care needs of refugees and asylum seekers. Medical schools have a social responsibility to integrate cultural competency into their curriculum and provide early exposure to working with marginalized groups. The E-Learning modules and CSL program allow medical students to gain experience working with marginalized populations, such as refugees, and consequently can contribute towards the development of culturally competent physicians.
